# Predicting and Analyzing Interactions between *Mycobacterium tuberculosis* and Its Human Host

**DOI:** 10.1371/journal.pone.0067472

**Published:** 2013-07-02

**Authors:** Holifidy A. Rapanoel, Gaston K. Mazandu, Nicola J. Mulder

**Affiliations:** Computational Biology Group, Department of Clinical Laboratory Sciences, Institute of Infectious Disease and Molecular Medicine, University of Cape Town, Cape Town, South Africa; Fundació Institut d’Investigació en Ciències de la Salut Germans Trias i Pujol, Universitat Autònoma de Barcelona, CIBERES, Spain

## Abstract

The outcome of infection by *Mycobacterium tuberculosis* (Mtb) depends greatly on how the host responds to the bacteria and how the bacteria manipulates the host, which is facilitated by protein–protein interactions. Thus, to understand this process, there is a need for elucidating protein interactions between human and Mtb, which may enable us to characterize specific molecular mechanisms allowing the bacteria to persist and survive under different environmental conditions. In this work, we used the interologs method based on experimentally verified intra-species and inter-species interactions to predict human-Mtb functional interactions. These interactions were further filtered using known human-Mtb interactions and genes that are differentially expressed during infection, producing 190 interactions. Further analysis of the subcellular location of proteins involved in these human-Mtb interactions confirms feasibility of these interactions. We also conducted functional analysis of human and Mtb proteins involved in these interactions, checking whether these proteins play a role in infection and/or disease, and enriching Mtb proteins in a previously predicted list of drug targets. We found that the biological processes of the human interacting proteins suggested their involvement in apoptosis and production of nitric oxide, whereas those of the Mtb interacting proteins were relevant to the intracellular environment of Mtb in the host. Mapping these proteins onto KEGG pathways highlighted proteins belonging to the tuberculosis pathway and also suggested that Mtb proteins might use the host to acquire nutrients, which is in agreement with the intracellular lifestyle of Mtb. This indicates that these interactions can shed light on the interplay between Mtb and its human host and thus, contribute to the process of designing novel drugs with new biological mechanisms of action.

## Introduction

Despite the wide variety of anti-tuberculosis drugs and significant progress made in treatment administration with improved control strategies in response to the global tuberculosis epidemic, tuberculosis (TB) remains an important health problem worldwide. According to the World Health Organization (WHO), 1.4 million people died from tuberculosis in 2010, 95% of them from the developing world. Currently, an effective vaccine is lacking [1], [2] and the existing tools for diagnosis and treatment of TB are limited. Furthermore, a growing prevalence of TB infection, co-infection with Human Immunodeficiency Virus (HIV) or Acquired Immunodeficiency Syndrome (AIDS) and an increased incidence of multi-drug resistant- (MDR-) and extensive drug resistent- (XDR-) TB constitute major impediments to global TB eradication programmes implemented so far. This shows that there is still a need for new drugs with novel therapeutic activities and vaccine to combat and prevent this disease.

As an intracellular pathogen, *Mycobacterium tuberculosis* Mtb, which causes TB, triggers mechanisms to ensure its growth and viability within the host. This happens through molecular interactions between specific pathogen proteins and host cells. As these molecular interactions influence molecular functions, they may allow the pathogen to alter its gene expression processes, to control the switching from a replicative (growth) to a non-replicative (dormancy) state and to develop alternative mechanisms for generating energy [2]. Thus, these interactions are essential for Mtb survival in the host by modulating the host response to bacterial infection or by acquiring nutrients it requires for its growth. This suggests that the identification of the protein interactions that Mtb uses to invade the host can contribute to the process of identifying potential targets for designing new drugs. Unfortunately, experimental studies of host-pathogen protein interactions are very scarce, so currently we have to rely on computational methods to elucidate human-Mtb interactions.

Studying protein-protein functional interactions allows the analysis of an organism's functioning as an integrated system and enables the identification of the patterns and properties driving systems. A functional interaction does not necessarily involve direct physical interaction or contact, but it rather refers to a relationship between proteins that contributes to cellular mechanisms through which a particular protein achieves its functions. The use of computational approaches and bioinformatics tools has opened a new route toward global analyses of whole genomes and investigating relationships between genes, providing the opportunity to look at genes within their context in the cell [3]. In the context of inter-species analysis, such as Mtb and its host, investigating functional interactions between genes that come into play when infection occurs may help elucidate mechanisms underlying virulence and pathogenesis associated with Mtb. Moreover, this would enhance our knowledge about this bacterial pathogen specific abilities for invasion and division inside host macropages, defeating the antibacterial mechanisms of these cells and resulting in disease in the host.

In this study, we predict human-Mtb functional interactions using the interologs method [4] based on intra-species and inter-species interactions. Thereafter, these interactions were subjected to different types of filters, yielding a total of 190 interactions that we overlaid onto a human and a previously generated Mtb strain CDC1551 protein functional interaction network [3], [5]. We conducted further analyses on these predicted interactions to determine whether these interactions are biologically feasible by looking at the subcellular location of interacting proteins. Functional analyses performed using the biological processes and pathways in which these interacting proteins are involved reveal that these interactions may help understand the interplay between the human and Mtb. This study provides insights into molecular mechanisms underlying the Mtb intracellular lifestyle in the host and host response.

## Results and Discussion

We used the literature to identify known human-Mtb interactions and exploited a computational approach, the interolog method, to predict inter-species interactions between human and Mtb. These interactions were filtered to ensure high confidence in the set of interactions produced, reducing the number of false positives. Subcellular location and functional analyses were conducted to elucidate biological relevance of these known and predicted interactions using Gene Ontology (GO) cellular component and biological process terms [6]–[8], and Kyoto Encyclopedia of Genes and Genomes (KEGG) pathways [9].

### Known Host–pathogen Interactions

Known human-Mtb interactions are not stored in databases as is the case for many human-virus protein interactions. Through manual curation of literature, we retrieved 47 inter-species interactions between Mtb and human, where both interacting proteins were present in the intra-species human and Mtb networks. The known human-Mtb protein interactions are mostly those involved in the recognition of the pathogen by the host. Specific host receptors recognize specific Mtb components. For example, the toll-like receptors (TLRs) recognize various bacterial lipoproteins (lprA, lpqH, lprG) whereas the human pulmonary surfactant protein A (PSP-A) binds to Mtb surface glycoproteins. In addition, the human plasminogen protein was found to be functionally related to 15 different bacterial plasminogen receptors.

### Predicted Interactions

The interolog method, described in the Materials and Methods section, was used to predict human-Mtb interactions and a total of 483 interactions between 175 human and 192 Mtb proteins were identified. Different filters were applied to assess these interactions, yielding three types of interactions: “interolog-DIP-known”, “interolog-DIP-array” and “interolog-HPI-array”, named after models used to infer and filter them. The two first types of interactions are derived using intra-species interactions retrieved from the Database of Interacting Proteins (DIP). Interolog-DIP-known interactions are filtered using the human-Mtb known interactions and interolog-DIP-array interactions are those filtered using expression data by considering only interactions where both interacting proteins are differentially expressed during infection. The interolog-HPI-array type of interactions are those inferred using inter-species interactions from experimentally verified Host-Pathogen Interactions (HPI) and filtered using expression data, as mentioned before. After applying these filters: three interactions involving 6 proteins were classified as “interolog-DIP-known”, 78 interactions between 35 human proteins and 47 Mtb proteins as “interolog-DIP-array” interactions and 109 interactions between 85 human proteins and 53 Mtb proteins as “interolog-HPI-array”. These different types of interactions are described below.

#### Interolog-DIP-known interactions

The interolog-known interactions comprise 3 interactions between 3 human proteins and 3 Mtb proteins, as shown in Table 1. We predicted that the human P07814 protein, which is a bifunctional aminoacyl-tRNA synthetase, interacts with P0A5U4 (RecA, recombinase A) from Mtb. The RecA protein is a recombinase functioning in recombinational DNA repair in bacteria. These two proteins are the neighbours of the human protein P00747 (plasminogen) and the mycobacterial protein P77899 (S-adenosylmethionine synthase), respectively, which are known to interact [10]. An interaction between the human protein ATP synthase subunit beta, mitochondrial, P06576 and the Mtb protein P0A548, a chaperone protein DnaJ 1, was also predicted. This second predicted interaction is the neighbour of three known interactions between one human protein (P00747) and three Mtb proteins: P0A5B9 (chaperone protein DnaK), P0A558 (elongation factor Tu) and P77899 (S-adenosylmethionine synthase). The third interaction involves the human protein P27361, which is a mitogen-activated protein kinase 3 (MAPK3), and the probable acetyl-CoA acyltransferase fadA2 from Mtb. MAPK3 is an enzyme which is a member of a MAPK family. Induction of the MAPK pathway is required for the expression of TNF-alpha, IL-10, and MCP-1 by human monocytes during Mtb infection [11]. The third interaction is the neighbour of the interacting proteins, the human protein O00206 (toll-like receptor 4) and the Mtb protein P0A520 (60 kDa chaperonin 2).

**Table 1:** The “interolog-DIP-known” interactions.

Human UniProt ID	Human protein name	Mtb UniProt ID	Mtb protein name
P07814	EPRS, bifunctional aminoacyl-tRNA synthetase	P0A5U4	RecA, recombinase A
P06576	ATP5B, ATP synthase subunit beta, mitochondrial	P0A548	DnaJ1, chaperone protein DnaJ 1
P27361	MAPK3, mitogen-activated protein kinase 3	O86361	fadA2, probable acetyl-CoA acyltransferase FADA2

#### Interolog-HPI-array and Interolog-DIP-array interactions

The Interolog-HPI-array interactions used experimentally verified inter-species interactions for the interolog predictions, which were further filtered using expression data. We predicted 109 interactions between 85 human proteins and 53 Mtb proteins. The network contains one large connected subnetwork of 61 proteins. In the largest subnetwork, we observe several proteins having 4 or more interactions. The human proteins NFKB1 and CD74 are connected to 10 and 5 Mtb proteins, respectively. Both of these proteins are known to play a role during TB infection. NFKB1 (nuclear factor NF-kappa-B subunit p105) is part of the NF-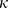
B complex which controls the transcription of genes involved in the pro-inflammatory response as well as genes involved in the antiapoptotic response. During early infection, Mtb inhibits macrophage apoptosis by up-regulating the NF-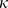
B signaling pathway, resulting in the up-regulation of FLIP, an inhibitor of death receptor signaling [12]. CD74 or HLA class II histocompatibility antigen gamma chain is implicated in the transport of MHC (major histocompatibility complex) class II proteins from the endoplasmic reticulum to the Golgi complex. Mtb inhibits MHC class II antigen presentation which reduces the recognition of infected macrophages by CD
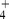
T cells [13]. The Mtb proteins HemL (glutamate-1-semialdehyde aminotransferase), RpoD (RNA polymerase sigma factor rpoD), RecN (DNA repair protein recN), ThiC (thiamine biosynthesis protein ThiC), CydD (Transmembrane ATP-binding protein ABC transporter) and SdaA (L-serine dehydratase) are connected to 10, 4, 8, 5, 5 and 4 human proteins, respectively.

**Figure 1:**
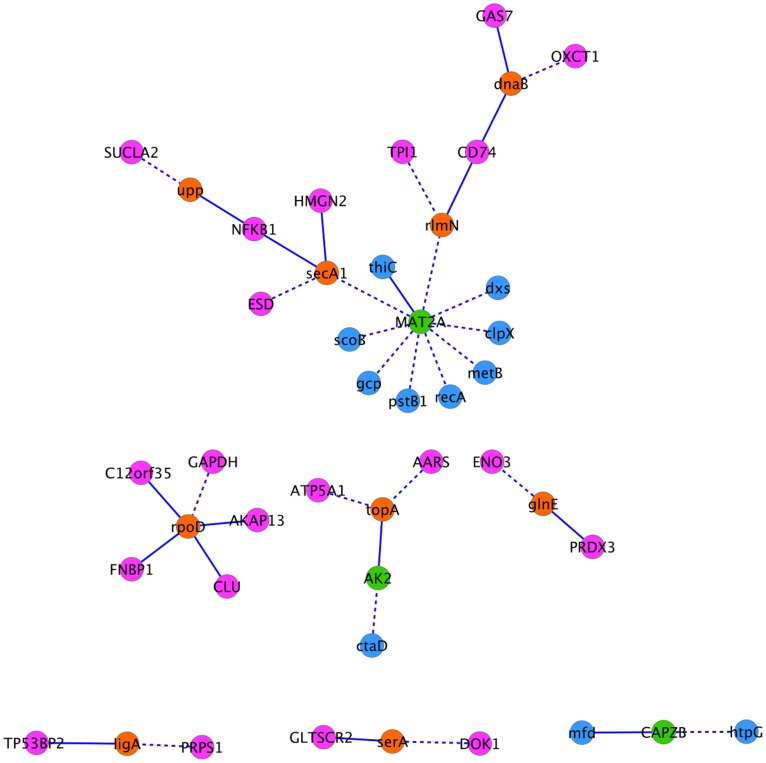
Common proteins between “interolog-DIP-array” and “interolog-HPI-array” interactions. Pink and blue nodes represent Mtb and human proteins, respectively. Orange nodes are the common Mtb proteins, and green nodes are the common human proteins. Dashed and solid lines are for “interolog-DIP-array” and “interolog-HPI-array” interactions, respectively.

The interolog-DIP-array interactions, consisting of 78 interactions between 35 human proteins and 47 Mtb proteins, were predicted by interologs using intra-species interactions and further filtered using expression data. There were no common interactions between the “interolog-DIP-array” and “Interolog-HPI-array” interactions, however, there were proteins shared by the two sets: 3 human proteins and 9 *Mtb* proteins (Figure 1). The shared human proteins are MAT2A (S-adenosylmethionine synthase isoform type-2), AK2 (Adenylate kinase 2, mitochondrial) and CAPZB (F-actin-capping protein subunit beta). The common *Mtb* proteins are DnaB (replicative DNA helicase), RlmN (Ribosomal RNA large subunit methyltransferase N), Upp (Uracil phosphoribosyltransferase), SecA1 (Protein translocase subunit SecA 1), RpoD (RNA polymerase sigma factor RpoD), TopA (DNA topoisomerase 1), GlnE (Glutamate-ammonia-ligase adenylyltransferase), LigA (DNA ligase) and SerA (D-3-phosphoglycerate dehydrogenase). Note that no overlap has been found between predicted and known interactions and this may be due to the independence of approaches used to infer these interactions.

### Cellular Component Analysis

Protein interactions are often constrained by physical location, meaning that two proteins are more likely to interact if they are in the appropriate subcellular location. Thus, we looked closely at the subcellular location of proteins involved in human-Mtb interactions to investigate possible occurrences of these interactions. Table 2 shows the subcellular locations of the interacting proteins for the different data sets.

**Table 2:** Number of proteins in each host-pathogen location pair.

Host protein location	Mtb protein location	Known	Interolog-DIP-known	Interolog-DIP-array	Interolog-HPI-array
Intracellular	Secreted	1	0	9	8
Intracellular	Cytoplasm	0	1	13	5
Intracellular	Cell membrane	0	1	23	35
Intracellular	Cell wall	0	0	22	11
Membrane	Cell membrane	5	0	1	23
Membrane	Cytoplasm	0	0	0	3
Membrane	Cell wall	2	1	5	4
Membrane	Secreted	10	0	1	1
Secreted	Cytoplasm	0	0	0	5
Secreted	Cell membrane	6	0	0	7
Secreted	Cell wall	3	0	0	0
Secreted	Secreted	21	0	0	1
Unknown	Cytoplasm	0	0	0	1
Unknown	Cell membrane	0	0	2	3
Unknown	Cell wall	0	0	2	2

Number of proteins in each host-pathogen location pair for proteins involved in the known, “interolog-DIP-known”, “interolog-DIP-array” and “interolog-HPI-array” interactions.

For the known host-pathogen protein interactions, the human proteins are located in the membrane or secreted; and the Mtb proteins are located in the cell membrane, cell wall or secreted. This is in agreement with the studies the interactions come from, which focus on the recognition of Mtb by human receptors which are located on the cell surface. The Mtb proteins are also located on the cell surface to enable binding to the human receptors. One exception is the interaction between the human heat shock protein 40 (HSP40) and the Mtb antigen mpt64. The human protein is annotated as intracellular and the bacterial protein is annotated as secreted, which is in accordance with [14], co-localizing mpt64 protein and HSP40 in the cytoplasm of HeLa cells.

**Figure 2:**
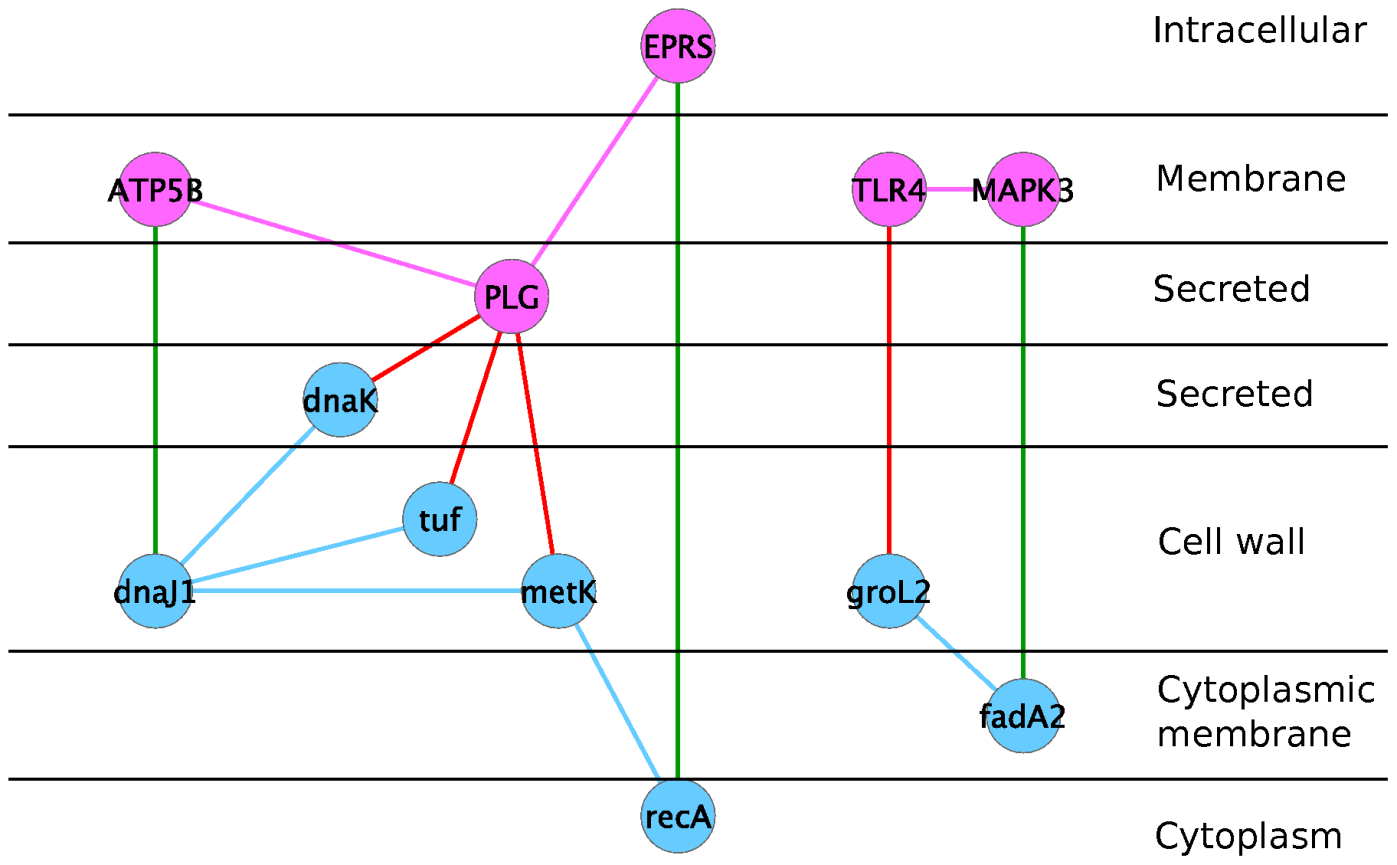
Interactions predicted by interologs which are neighbours of known interactions. Pink nodes are human proteins and pink edges are human interactions. Blue nodes are Mtb proteins are blue edges are Mtb interactions. Red edges are known human-Mtb interactions and green edges are inter-species interactions predicted by interologs.

Mtb is an intracellular pathogen that has evolved strategies to survive in intracellular phagosomes. A study by van der Wel *et al.* [15] shows that after two days of infection, Mtb progressively translocates from phagolysosomes into the cytosol in nonapoptotic cells. Therefore, the likely locations of the host-pathogen protein pairs are intracellular-cell-wall/cell-membrane/secreted if the pathogen is within the host cell, or secreted-cell-wall/cell-membrane/secreted if the pathogen just encounters the host cell. Figure 2 displays locations of interolog-known interactions listed in Table 1, together with their neighours belonging to known interactions.

Note that, although GO and PSORTb predicted some of the interacting Mtb proteins to be cytoplasmic, TubercuList annotation (http://genolist.pasteur.fr/TubercuList) for some proteins, for example ScoB, Glpk and GadB, suggests that these proteins have been identified by mass spectrometry in the membrane fraction of Mtb cells. Therefore, while the subcellular location analysis provides us with more biological insight into the predicted interactions, we did not want to rule out possible interactions based on subcellular location predictions.

### Gene Ontology Term Enrichment

To gain insight into the biological processes of the proteins involved in human-Mtb interactions, we performed a GO term enrichment analysis using the Database for Annotation, Visualization and Integrated Discovery (DAVID) Functional Annotation Chart tool [16], [17]. For each protein list of interest (“interolog-DIP-array” or “interolog-HPI-array” proteins), we performed a GO biological process enrichment analysis using the human proteins or Mtb proteins from our constructed network as the background. As suggested on the DAVID website, we selected the GO terms situated in the fourth and fifth level to avoid very general GO terms such as “biological process”. We used the Bonferonni 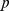-value, which is a corrected 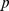-value for multiple testing, and we selected those GO terms enriched in our candidate protein list by requiring a 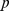-value less than 0.05.

The human “interolog-DIP-array” proteins predicted to interact with Mtb proteins were enriched in nitrogen compound biosynthetic process, oxoacid metabolic process, carboxylic acid metabolic process and nucleobase, nucleoside and nucleotide metabolic process as shown in Table 3. When Mtb cells are phagocytozed they are exposed to nitric oxide and oxidative stress. The interacting human proteins may be involved in facilitating this. On the other hand, the human “interolog-HPI-array” proteins are enriched in processes related to negative regulation of apoptosis and positive regulation of cellular process (see Table 3). It is known that virulent Mtb strains inhibit apoptosis of the host macrophage to protect their replicative niche [18].

**Table 3:** Enriched GO biological process terms.

GO Id	GO term	Number of genes	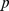 -values	Bonferonni-correction
GO:0044271	Nitrogen compound biosynthetic process	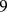	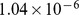	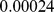
GO:0043436	Oxoacid metalic process	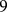	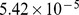	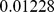
GO:0019752	Carboxylic acid metalic process	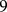	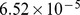	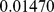
GO:0055086	Nucleobase, nucleoside and nucleotide metabolic process	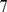	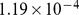	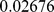
GO:0048522	Positive regulation of cellular process	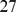	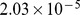	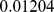
GO:0042981	Regulation of apoptosis	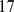	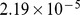	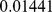
GO:0043067	Regulation of programmed cell death	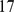	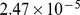	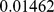
GO:0010941	Regulation of cell death	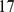	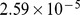	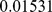
GO:0043066	Negative regulation of apoptosis	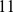	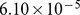	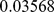
GO:0043069	Negative regulation of programmed cell death	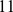	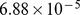	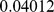
GO:0060548	Negative regulation of cell death	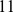	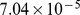	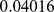

Enriched GO biological process terms in human “interolog-DIP-array” proteins (top) and “interolog-HPI-array” proteins (bottom).

From the Mtb side, the smallest Bonferonni corrected 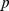-values were approximately 0.10032 and 0.34548 for GO biological process terms of proteins involved in “interolog-DIP-array” and “interolog-HPI-array” interactions, respectively. This indicates that there is no sufficient evidence that our gene or protein list is enriched in any GO biological processes. However, when looking at the individual Mtb proteins involved in these interactions, some of them share the same biological processes with Mtb proteins previously known to interact with human. These proteins and their GO terms are listed in Table 4. The biological processes include those particularly relevant to the intracellular environment of Mtb in the host, such as growth of symbiont in host, response to stresses related to the harsh intracellular environment, response to host immune response, and pathogenesis. Proteins playing a role in these processes may achieve their goal in protecting the pathogen from the environment through interaction with host proteins.

**Table 4:** Important GO biological processes of Mtb “interolog-DIP-array” proteins (top) and “interolog-HPI-array” proteins (bottom).

UniProt Acc	Protein name	GO ID	GO name
O53832	PstB1	GO:0035435	phosphate ion transmembrane transport
		GO:0044117	growth of symbiont in host
P0A558	Tuf	GO:0001666	response to hypoxia
		GO:0006184	GTP catabolic process
		GO:00100039	response to iron ion
P0A602	RpoD	GO:0009405	pathogenesis
		GO:0009415	response to water
		GO:0052572	response to host immune response
P63288	ClpB	GO:0006950	response to stress
		GO:0009408	response to heat
P63650	ScoB	GO:0001666	response to hypoxia
P63852	CtaD	GO:0009060	aerobic respiration
P64411	HtpG	GO:0006950	response to stress
		GO:0071451	cellular response to superoxide
P69942	GlnE	GO:0040007	response to ammonium ion
O53306	FadD13	GO:0001101	response to acid
		GO:0044119	growth of symbiont in host cell
P63393	IrtB	GO:0009405	pathogenesis
O53189	Tig	GO:0006457	protein folding
		GO:0009267	cellular response to starvation
		GO:0046677	response to antibiotic
O06559	NarG	GO:0001101	response to acid
		GO:0001666	response to hypoxia
P95095	CstA	GO:0009267	cellular response to starvation
P66014	RelA	GO:0009405	pathogenesis
P71717	MbtB	GO:0009405	pathogenesis
		GO:0052572	response to host immune response
Q50723	Rv3402c	GO:0052572	response to host immune response
P0A602	RpoD	GO:0009405	pathogenesis
		GO:0052572	response to host immune response

Furthermore, 32 and 22 proteins from the “interolog-DIP-array” and the “interolog-HPI-array” Mtb lists, respectively, were among the essential genes required for growth identified by Sassetti *et al.* using transposon site hybridization (TraSH) [19]. The genes in Sassetti *et al.* were initially for Mtb H37Rv but the orthologue file from the Integr8 project [20] allowed us to map them to CDC1551 genes. In particular, 3 “interolog-HPI-array” proteins are also virulence factors of Mtb, namely NarG (O06559), RelA (P66014) and MbtB (P71717). NarG is a nitrate reductase. Nitrate respiration helps the bacteria to survive in O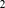-depleted areas of inflammatory or necrotic tissue. NarG interacts with the human protein sorting nexin SNX6 (Q9UNH7), which is thought to be involved in several stages of intracellular trafficking. RelA is a protein that coordinates the metabolism of (p)ppGpp, a mixture of 3′-pyrophosphate derivative of GDP (ppGpp) and 3′-pyrophosphate derivative of GTP (pppGpp). Under stress conditions, such as nutrient starvation, RelA produces (p)ppGpp that accumulates intracellularly and suppresses synthesis of stable RNA, induces degradative pathways and modulates expression of genes involved in DNA replication [21]. RelA was predicted to interact with the human protein CALCOCO1 (Q9P1Z2). CALCOCO1 is thought to be involved in elementary cellular functions linked to Ca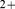/calmodulin signaling [22]. Finally, MbtB, also known as mycobactin synthetase protein B, is involved in the initial steps of the mycobactin biosynthetic pathway. Mycobactins are lipophilic siderophores of mycobacteria mediating iron acquisition within macrophages [23]. MbtB interacts with the human proteins stabilin-1 STAB1 (Q9NY15) and cofilin-1 CFL1 (P23528). Stabilin-1 is a scavenger receptor and binds to both Gram-positive and Gram-negative bacteria [24] whereas cofilin-1 is a 18kDa phosphoprotein that regulates actin cytoskeleton dynamics.

### Pathways

Bacterial pathogens attack intracellular-signalling and cytoskeletal pathways to alter host responses in a way that benefits them. For example, Mtb interferes with the NF-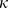
B and the MAPK signalling pathways [25] leading to the prevention of NF-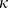
B dependent transcription and the alteration of antigen presentation, respectively. Bacteria might also hijack host metabolic pathways. For instance, *Chlamydia pistacci* hijacks the host's tryptophan depletion pathway by intercepting the byproduct kynurenine, which is used by *C. pistacci* to produce its own tryptophan [26]. We used the Kyoto Encyclopedia of Genes and Genomes (KEGG) [9] to find pathways to which proteins involved in predicted Human-Mtb interactions belong. The KEGG Mapper-Search and Color Pathway mapping tool allows us to search given objects, such as genes, proteins or compounds against KEGG pathway maps. For each list of proteins (human and Mtb, “interolog-DIP-array” or “interolog-HPI-array”), we obtained a list of pathways relevant to the protein list. However, not all proteins belong to a pathway and a protein may be part of several pathways.

**Figure 3:**
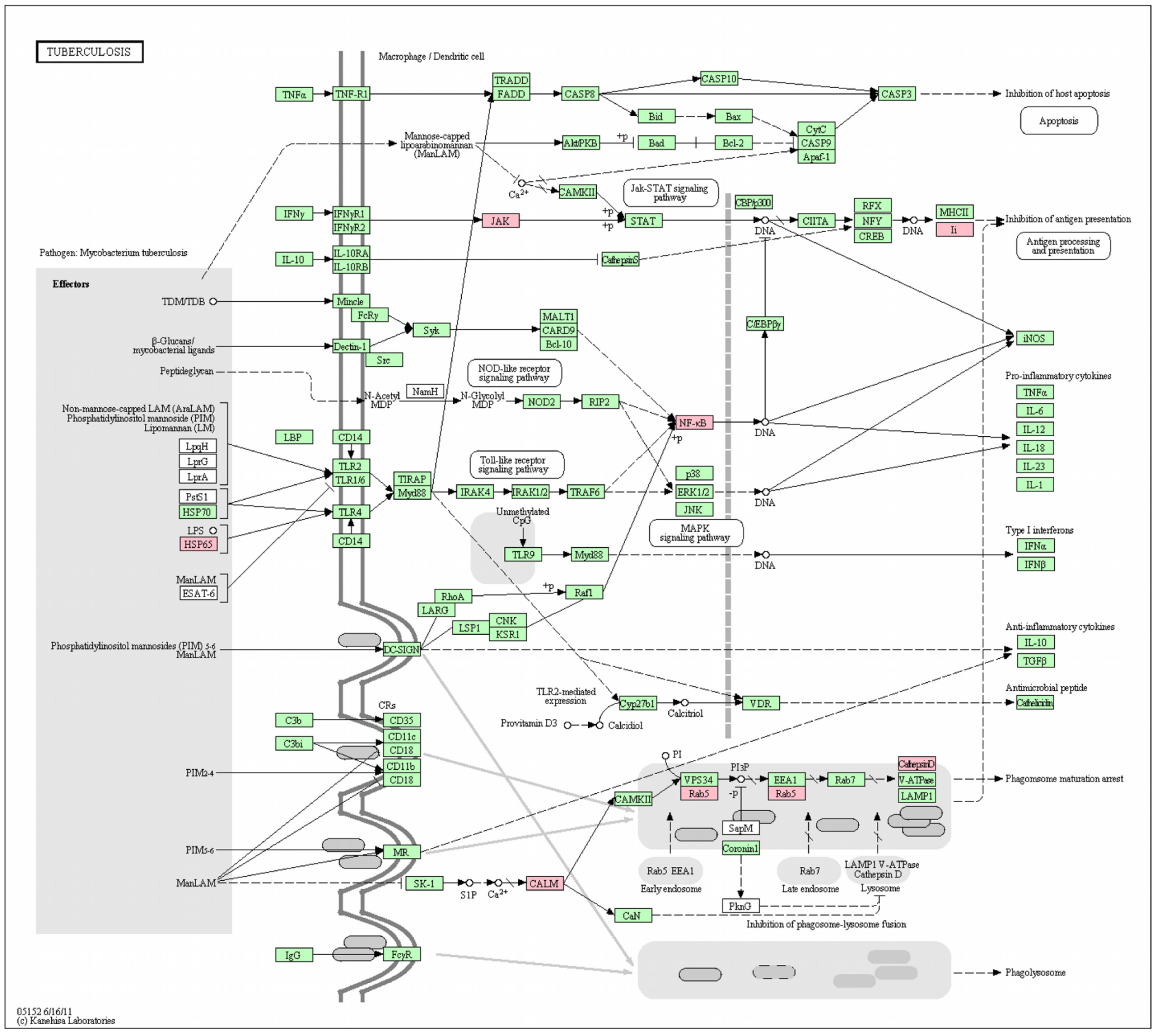
The “interolog-HPI-array” proteins overlaid on the tuberculosis pathway from KEGG. Pink background represents the proteins present in the “interolog-HPI-array” list.

Interestingly, the first pathway retrieved while searching the human “interolog-HPI-array" proteins against KEGG is the tuberculosis pathway. 8 proteins, namely CTSD, HSPD1, JAK1, NFKB1, RAB5A, RAB5C, CALM1 (P62158) and CD74, were mapped onto the tuberculosis pathway. In particular, CTSD, RAB5A, RAB5C and CALM1 are all involved in phagosome maturation which is blocked by Mtb. CALM1 is a Ca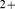-dependent effector protein necessary for the activation of CaMKII, which is required for the recruitment of EEA1 to the phagosome. RAB5A and RAB5C are GTPases necessary for phagosome maturation through the recruitment of a large number of effector proteins. Lastly, CTSD or cathepsin D is a hydrolytic protease secreted by the phagolysosome for bacterial degradation. Figure 3 represents the proteins highlighted on the tuberculosis pathway from KEGG.

While searching the Mtb proteins from “interolog-DIP-array” or “interolog-HPI-array” against KEGG, one protein from “interolog-DIP-array”, namely GroEL (P0A520), was found to belong to the tuberculosis pathway. GroEL (HSP65, highlighted in Figure 3) is a 60 kDa chaperonin 2 protein known to interact with TLR4. In the “interolog-array” data, it was predicted to interact with three human proteins: CLPP (Q16740), a putative ATP-dependent Clp protease proteolytic subunit, mitochondrial, TUFM (P49411), an elongation factor Tu, mitochondrial, and PRPS2 (P11908), which is a ribose-phosphate pyrophosphokinase 2. Even if the other Mtb proteins did not map to the tuberculosis pathway, some of them have biological processes that are present in the list of known Mtb proteins interacting with human proteins (Table 4). Metabolic pathways, biosynthesis of secondary metabolites and microbial metabolism in diverse environments are the top three pathways the Mtb “interolog-DIP-array” and “interolog-HPI-array” proteins map to.

**Figure 4:**
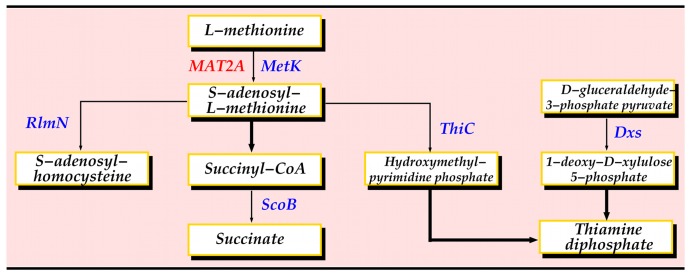
Interactions involving MAT2A. A thin arrow indicates direct interaction, whereas a thick arrow indicates that there are several steps.

For each human-Mtb protein interaction with both partners belonging to one or more pathways, we looked for common pathways, substrates or products, as well as pathways involved in TB infection. By applying this method, we were able to reconstruct a small network centered on the human MAT2A protein (Figure 4). MAT2A catalyzes the reaction from L-methionine to S-adenosyl-L-methionine in cysteine and methionine metabolism. MAT2A was predicted to interact with 10 Mtb proteins in total. 3 of these proteins, namely MetK, RlmN and ThiC, have direct links to L-methionine or S-adenosyl-L-methionine in metabolic pathways. MetK is a methionine adenosyltransferase and performs the same function as MAT2A. RlmN is a ribosomal RNA large subunit methyltransferase N and specifically methylates position 2 of adenine 2503 in 23S rRNA. Finally, ThiC is a hydroxymethylpyrimidine phosphate synthase catalyzing the synthesis of the hydroxymethylpyrimidine phosphate (HMP-P) moiety of thiamine from aminoimidazole ribotide (AIR) in a radical S-adenosyl-L-methionine dependent reaction. ThiC is an essential gene necessary for thiamin (vitamin B1) synthesis [27]. Two proteins interacting with MAT2A, namely Dxs and ScoB, belong to pathways using S-adenosyl-L-methionine. Dxs catalyses the formation of 1-deoxy-D-xylulose 5-phosphate, which is consumed during the biosynthesis of the thiazole moiety of thiamin. ScoB is the B subunit of probable succinyl-CoA:3-ketoacid-coenzyme A transferase.

### Predicted Interactions and Drug Targets

One of the main reasons to study host-pathogen protein interactions is the possibility of finding targets to design new drugs. This is particularly important in the case of TB because of the existence of drug resistance. A list of 881 potential drug target proteins was computationally predicted for the Mtb network in a separate study in the laboratory based on their network properties [3]. These are important proteins in the Mtb functional network since they are responsible for several indirect functional connections between other proteins in network. 878 out of 881 proteins were present in the Mtb network from this study. We used this list to identify potential drug targets in the Mtb proteins predicted to interact with human proteins and we used the Fisher's exact test to determine whether the predicted list of proteins contains more drug targets than expected by chance.

**Figure 5:**
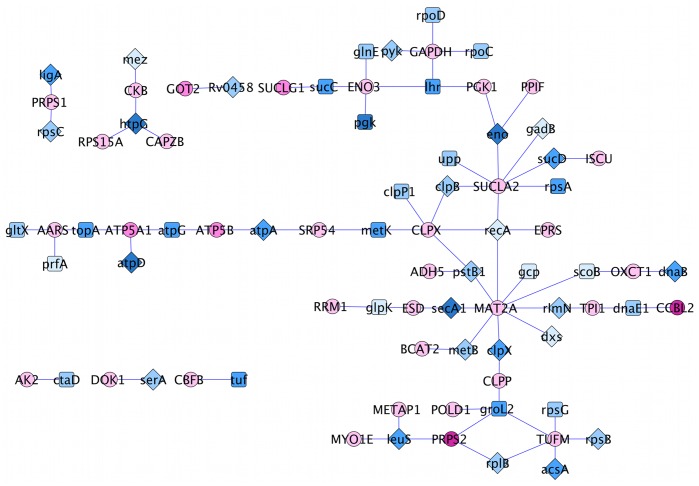
Drug targets in the “interolog-DIP-array” network are depicted with a diamond shape. Pink and blue nodes represent Mtb and human proteins, respectively. The color grades reflect protein subcellular localizations, the more we move outside the cell, the darker the color.

**Figure 6:**
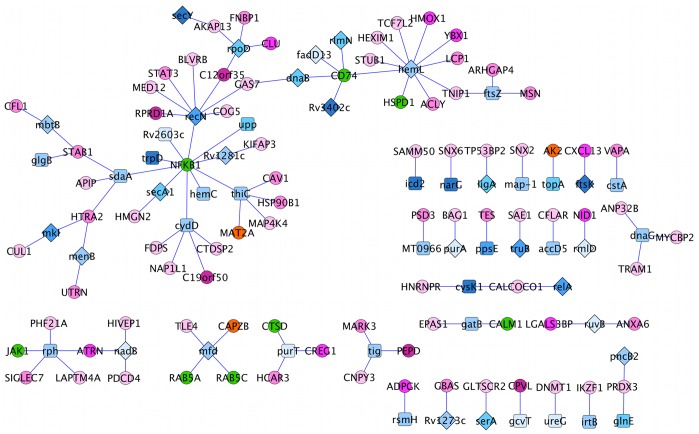
Drug targets in the “interolog-HPI-array” network are depicted with a diamond shape. Pink and blue nodes represent Mtb and human proteins, respectively. The color grades reflect protein subcellular localizations, the more we move outside the cell, the darker the color.

Table 5 presents the number of potential drug targets in each Mtb protein list with the 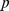-value obtained using the Fisher's exact test. Taking a cut-off of 0.05 for the 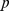-value, the “interolog-DIP-array” and “interolog-HPI-array” protein lists contain more drug targets than would be expected by chance, with 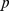-values of 2.41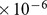 and 3.80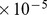, respectively. Figures 5 and 6 highlight the Mtb drug target proteins in the “interolog-DIP-array” and “interolog-HPI-array” interactions, respectively. 5 of the human proteins from the “interolog-HPI-array” interactions that mapped onto the tuberculosis pathway interact with Mtb proteins predicted to be drug targets. In particular, 4 Mtb proteins interacting with CD74 are predicted drug targets.

**Table 5:** Number of drug targets and 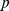-value for each Mtb protein list.

Mtb protein list	Number of drug targets	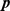 -value
Interolog-known	1/3	0.52
Interolog-array	25/47	2.41×10^−6^
Database-array	25/53	3.80×10^−5^

### Conclusions

In this work, we have predicted and analyzed human-Mtb functional interactions to gain more insights into molecular mechanisms underlying the communication flow between *Mycobacterium tuberculosis* (Mtb) and human during infection. After filtering the set of predicted interactions, for those with evidence of expression during infection, we first looked at the subcellular locations of the proteins involved in the predicted interactions to assess whether these predicted interactions are biologically feasible. We observed that the Mtb proteins were mostly located on the cell surface, whereas human proteins were mostly tagged as intracellular, showing the intracellular location of the bacteria in the host. These subcellular locations reflect the fact that the bacteria is located within the host cells and suggests that these predictions are potentially feasible.

We performed functional analyses based on biological processes and pathway maps in which the interacting proteins are involved. The biological process analysis of these proteins suggests that human proteins participating in these interactions play a crucial role in facilitating the production of nitric oxide and in negative regulation of apoptosis. The biological processes of Mtb proteins include those particularly relevant to the intracellular environment of Mtb in the host. Thus, these interactions illustrate how Mtb might acquire nutrients and how it modulates the host response to its advantage. Mapping the predicted interactions onto KEGG pathways revealed that some of the proteins are known to play a role in the “tuberculosis” pathway and that Mtb might hijack the host to acquire nutrients. We also found that the predicted Mtb proteins are enriched in predicted drug targets. Thus, such a study can help us to understand the interplay between the host and pathogen and may prove useful for identifying new drug targets.

## Materials and Methods

The human functional interaction network was constructed by combining three datasets, namely the human protein interaction network used by Bossi and Lehner [28], together with data from the REACTOME [29] and STRING [30], [31] databases. Using a cut-off score of 0.65, a human network of 16,548 proteins and 334,070 interactions was obtained. This cut-off score was chosen so that the interactions in the Bossi and Lehner network which have a score greater than or equal to 0.65 and are considered reliable could be integrated into the network. A previously generated functional network was used for Mtb [3], [5], which combines data extracted from the STRING database, gene expression and sequence data [32]–[34]. The final Mtb network contains 4,070 proteins and 38,049 interactions. Note that here we used a stricter cut-off score for selecting interactions compared to [3], [5].

### Predicting Host-pathogen Interactions

**Figure 7:**
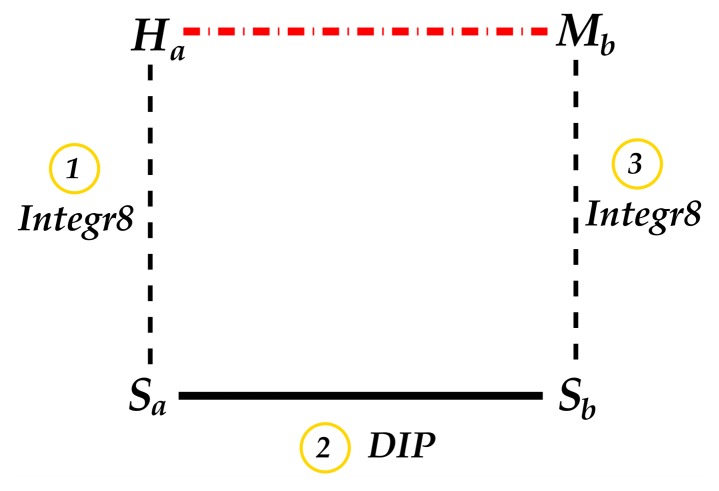
Steps for inferring inter-species interactions using the interologs method. Dotted lines represent orthologs, the solid line represents intra-species interaction and the dashed line represents inferred inter-species interaction.

**Figure 8:**
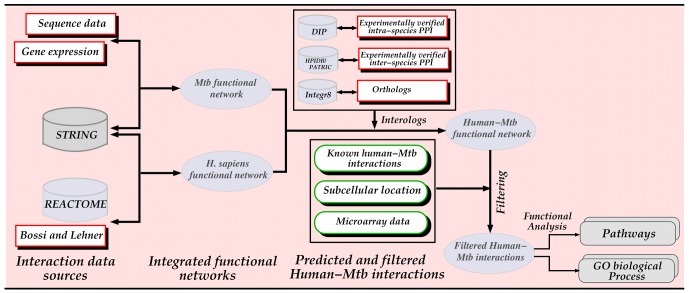
Flowchart depicting the prediction of host–pathogen interactions and their analysis.

The interologs method was used to infer host-pathogen interactions. Interologs are conserved interactions between a pair of proteins which have interacting orthologs in another organism [4]. More precisely, the interaction 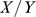 in one species is referred to as interologs of 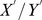 in another species if 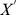 and 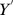 are orthologs of 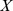 and 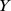, respectively. The ortholog data was retrieved from the Integr8 project [20] at the European Institute of Bionformatics (EBI) and intra-species interactions are from the Database of Interacting Proteins (DIP) [35] as depicted in Figure 7. Note that these inter-species functional interactions were further investigated by filtering based on two criteria: connections to known interactions, referred to as “interolog-DIP-known” interactions, and whether they are expressed during infection, referred to as “interolog-DIP-array” interactions. The procedure followed is shown in Figure 8.

### Filtering of Predicted Host-pathogen Interactions

#### Interolog-DIP-known dataset

Given a known human-Mtb interaction and an interaction predicted by interologs using interaction data downloaded from the DIP database, if the human interolog protein is a neighbour of the human known interacting protein and the Mtb interolog protein is a neighbour of the Mtb known interacting protein, then the interaction predicted by the interolog is an “interolog-DIP-known” interaction. Note that the neighbours of a protein are the proteins directly connected to it in the protein interaction network. This was applied to all possible (x,y) pairs, where x is a human-Mtb interaction predicted by interologs and y is a known human-Mtb interaction, to produce the set of “interolog-DIP-known” interactions. Note that human or Mtb known protein refers to human or Mtb protein involved in a known human-Mtb interaction and similarly, human or Mtb interolog protein is a protein involved in an interaction predicted by interlogs.

#### Interolog-DIP-array dataset

The outcome of an infection depends on how the host responds to the pathogen and how the pathogen evades the immune system. Investigation of the entire transcriptome of a cell during infection may provide a hint about the host-pathogen cross-talk. This is feasible using microarray technology which allows the simultaneous analysis of expression of thousand of genes. Therefore, this method has been widely used to study the interplay between the host and the pathogen. Unfortunately, the microarray data gives us only a set of genes that might interact as they are expressed under the appropriate conditions, but does not tell us explicitly which gene interacts with which other gene. That is why the interactions predicted by interologs were combined with the microarray data: the interologs predicts the interactions and the microarray data ensures that the interactions could occur as the interactors are present during infection. Microarray data studying the transcriptional changes upon infection both in Mtb and in macrophages and dendritic cells derived from the same donors over a time-course experiment from Tailleux *et al.* [36] was used for the analysis. Raw human microarray data used in our analysis was provided by the authors of the article. We performed the analysis described in [36] on the data to obtain the human genes differentially expressed during Mtb infection. In this paper, the authors applied a filter based on Detection calls to filter out noisy data before selecting differentially expressed genes: they first removed the probe sets called “Absent” over all conditions and replicates; then they determined the 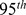 percentile of all the signals of the entire dataset flagged with an absent call and used it as a threshold to remove all the remaining probe sets whose expression values were always below the threshold in each sample. The remaining probe sets were used for the analysis. Differentially expressed genes were detected using the Limma Bioconductor library, which is based on the fitting of a linear model to estimate the variability in the data. A threshold p-value of 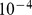 was used to select differentially expressed genes. The list of Mtb genes differentially expressed were downloaded from B
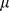
G@Sbase under the accession E-BUGS-58 (http://bugs.sgul.ac.uk/bugsbase). To find the “interolog-array” interactions, we took the interologs and found those where both the human and Mtb protein partners were differentially expressed in the above experiments.

#### Interolog-HPI-array dataset

Here, we used a variant of the interologs method, referred to as “Interolog-HPI-array”, where instead of using intra-species interactions to predict the interologs, experimentally verified interactions between human and bacterial proteins were used. From the inter-species human-pathogen interactions, if the pathogen protein has an ortholog in Mtb, an interaction between the human and Mtb proteins was inferred. The experimentally verified host-pathogen interactions were downloaded from two databases dedicated to inter-species interactions, namely the Pathosystems Resource Integration Center (PATRIC) [37] and the Host-Pathogen Interaction database (HPIDB) [38]. We filtered host-pathogen interactions from HPIDB to only retrieve human-bacterial protein interactions. All the protein interactions from PATRIC were used since it contains only human-bacterial protein interactions. These interologs were then filtered by taking the interactions where both proteins are differentially expressed during infection. The same microarray data as for the “interolog-DIP-array” interactions was used.

### Subcellular Localization

Gene ontology (GO) cellular component (GO CC) terms were used to find the location of the human and *Mtb* proteins. The GO CC terms were converted to GO slim terms. A GO slim is a truncated version of the GO ontologies where the more specific terms are mapped to one or more high-level terms. The “Investigate GO slim option” of the QuickGO tool from EBI was used for that purpose.

For the human proteins, we used the GO slim generic, which is a predefined GO slim set. The set is not species specific but it has many terms pertaining to eukaryotic organisms, and contains 35 cellular components terms. We used all the protein IDs of the human network to filter the annotation. We then downloaded the results and collected the GO cellular components that we divided into three groups: intracellular, membrane and secreted. We also have the unknown category containing proteins that have no cellular component annotation. The intracellular group contains the terms referring to all components inside of the cells. The membrane group only contains the plasma membrane and the secreted group contains the proteins in the extracellular region. Since a protein may be annotated to more than one group, we set the final annotation of the protein as the furthest it can go outside the cell.

We used only the GO slim terms relevant to prokaryotes in the GO slim generic to build the GO slim terms for the Mtb proteins and we used all the protein IDs of the Mtb network to filter the annotation. In the case where the protein had no subcellular location, we used PSORTb to predict its location, which was the case for 2,272 proteins. PSORTb is a web-based tool for prediction of subcellular location in bacterial protein sequences [39]. The final predictions of localization of proteins were “Cytoplasmic”, “CytoplasmicMembrane”, “Cellwall”, “Extracellular” and “Unknown”. We used these locations to divide the predicted locations into 5 main categories, from the inside of the cell to the outside: cytoplasm, cell membrane, cell wall, secreted and unknown. Secreted proteins are proteins secreted into the cell surroundings and proteins whose locations could not be identified were classed as “unknown”. Again, if a protein was annotated with more than one location, then we took the one that is furthest from the inside of the cell.

### Human-Mtb Protein Interaction Functional Analysis

To understand the functional features of the predicted human-Mtb interactions, we are interested in the biological processes in which interacting proteins are involved, as well as in the pathway maps to which they belong in order to characterize the biological role of these interactions. For this purpose, we used the known human and Mtb protein annotation data downloaded from the Gene Ontology Annotation (GOA) project (http://www.ebi.ac.uk/goa) and a list of pathways relevant to the protein list from the KEGG database. We used the DAVID tool to perform a GO biological process term enrichment analysis in order to highlight the most relevant terms considering proteins in the intra-species network generated as the background. The DAVID tool was initialized to select GO terms located from the fourth or fifth levels of the GO directed acyclic graph to avoid very general GO terms.
